# A Web-Based Therapeutic Program (We Can Do This) for Reducing Methamphetamine Use and Increasing Help-Seeking Among Aboriginal and Torres Strait Islander People: Protocol for a Randomized Wait-List Controlled Trial

**DOI:** 10.2196/14084

**Published:** 2019-07-26

**Authors:** Rachel Reilly, Rebecca McKetin, Handan Wand, Julia Butt, Matthew Smout, Nadine Ezard, Katherine Conigrave, Yvonne Clark, Brendan Quinn, Carla Treloar, Dennis Gray, Adrian Dunlop, Yvette Roe, James Ward

**Affiliations:** 1 Infectious Diseases Aboriginal Health Aboriginal Health Equity South Australian Health and Medical Research Institute Adelaide Australia; 2 College of Medicine and Public Health Flinders University Bedford Park Australia; 3 National Drug Research Institute Curtin University Perth Australia; 4 Kirby Institute University of New South Wales Sydney Australia; 5 School of Psychology, Social Work and Social Policy University of South Australia Adelaide Australia; 6 National Centre for Clinical Research on Emerging Drugs St Vincent’s Hospital Sydney Australia; 7 University of New South Wales Sydney Australia; 8 Drug Health Services Royal Prince Alfred Hospital Camperdown Australia; 9 Discipline of Addiction Medicine University of Sydney Camperdown Australia; 10 Aboriginal Families Health Research Partnership South Australian Health and Medical Research Institute Adelaide Australia; 11 Australian Institute of Family Studies Melbourne Australia; 12 Centre for Social Research in Health Social Policy Research Centre University of New South Wales Sydney Australia; 13 School of Medicine and Public Health University of Newcastle Newcastle Australia; 14 Drug and Alcohol Clinical Services Hunter New England Health Newcastle Australia; 15 Molly Wardaguga Research Centre Charles Darwin University Darwin Australia

**Keywords:** methamphetamine, eHealth, Australian Aboriginal people, Aboriginal health services

## Abstract

**Background:**

Methamphetamine use is of deep concern to Aboriginal and Torres Strait Islander communities, but access to culturally appropriate treatment resources and services is limited. Web-based programs have potential as flexible and cost-effective additions to the range of treatment options available to Aboriginal people. The We Can Do This online intervention is designed to incorporate evidence-based therapies in a culturally relevant format using narratives from Aboriginal people to contextualize the therapeutic content.

**Objective:**

The goal of the research will be to test the effectiveness of the online intervention in a wait-list controlled randomized trial across multiple sites in urban, regional, and remote locations.

**Methods:**

Participants will be Aboriginal and Torres Strait Islander people aged 16 years and over who have used methamphetamine at least weekly for the previous 3 months. They will be recruited online and via health services. During the intervention phase, participants will have access to the online intervention for 6 weeks with optional telephone or face-to-face support provided by participating health services. The primary outcome measure will be the number of days the participant used methamphetamine over the past 4 weeks compared to wait-list controls, assessed at baseline, 1, 2, and 3 months. Secondary outcomes will include help-seeking, readiness to change, severity of dependence, and psychological distress. Any important changes to the protocol will be agreed upon by the trial management committee and communicated to all relevant parties, including trial site representatives and the trial registry.

**Results:**

Recruitment will commence in July 2019, and results are expected in early 2021. This research is funded by National Health and Medical Research Council project grant #1100696. The primary sponsor for the trial is the South Australian Health and Medical Research Institute. A trial management committee with representation from the participating health services, chief investigators, other Aboriginal experts, and consumers will oversee procedures, trial conduct, analysis, and reporting of the results.

**Conclusions:**

The trial of this online intervention builds on existing research supporting the effectiveness of Web-based therapies for a range of psychological and other health-related issues including substance use. If successful, the We Can Do this online intervention will increase the range of options available to Aboriginal people seeking to reduce or stop methamphetamine use. It may provide a pathway into treatment for people who may otherwise be disengaged with health services for a range of reasons and will be a culturally appropriate, evidence-based resource for health practitioners to offer their clients.

**Trial Registration:**

Australian New Zealand Clinical Trials Registry ACTRN12619000134123p; https://www.anzctr.org.au/ Trial/Registration/TrialReview.aspx?id=376088&isReview=true

**International Registered Report Identifier (IRRID):**

PRR1-10.2196/14084

## Introduction

### Background

Methamphetamine use has increased globally over recent years [1]. In Australia, where amphetamine-type stimulants have been a feature of the illicit drug market for many decades, a shift from low-purity powder (speed) to the typically higher purity form of the drug, crystalline methamphetamine (often referred to as ice), has led to an increase in drug-related harms [2,3]. This is indicated by increases in amphetamine-related hospital admissions, drug and alcohol service treatment presentations, and helpline calls [4]. The available evidence indicates that Aboriginal and Torres Strait Islander Australians (hereafter, Aboriginal) use methamphetamine and experience harms at a higher rate than non-Indigenous Australians, with some of the highest recorded rates of use in regional and remote areas [5,6].

In line with national trends, Aboriginal Community Controlled Health Services (ACCHSs) have experienced an increase in amphetamine-type stimulant-related presentations over recent years. The burden of service provision for amphetamines is noted in the 2014-2015 Aboriginal and Torres Strait Islander Health Organizations Online Service Report, where the proportion of organizations that reported amphetamine as one of their most important issues, in terms of staff time and organizational resources, increased from 45% in 2013-2014 to 70% in 2014-2015. At the same time, social and emotional well-being (incorporating mental health within a unique set of culturally informed well-being domains [7]) and substance use services were identified as among the top five service gaps, and access to evidence-based treatments provided by qualified staff remains variable [8].

A systematic review of psychological treatments applied specifically to methamphetamine use concluded that good clinical outcomes are achieved with cognitive-behavioral therapy (CBT) [9]. Further, CBT with or without motivational interviewing appears to be associated with reductions in methamphetamine use and other positive changes [9]. A preliminary randomized controlled trial comparing acceptance and commitment therapy (ACT) and CBT indicated that these two therapeutic modalities produce comparable results; therefore, ACT may be a viable alternative to CBT for treating harmful methamphetamine use [10]. This is relevant in an Aboriginal context where written, worksheet-based CBT approaches are not always preferred for a range of reasons including literacy and a cultural preference for approaches incorporating yarning and story-telling [11].

Internet interventions have shown effectiveness for some substance use problems, including methamphetamine use. For example, Breaking the Ice was a fully automated, participant self-guided, Web-based intervention consisting of six self-administered modules incorporating components of CBT and motivational interviewing [12]. The program was effective at increasing help-seeking and participant motivation to reduce their methamphetamine use and reducing days out of role, relative to a wait-list control (at 3 to 6 months from baseline). Effect sizes were small to moderate (relative risk 0.5-0.74) [13]. The program participants who completed the Web-based modules, predominantly non-Aboriginal and male, showed a nonsignificant reduction in their methamphetamine use [13]. Feedback on the acceptability of Breaking the Ice was generally positive in relation to the use of fictional case stories as an engaging approach, but participants reported that the program was also too text-heavy, which may have contributed to attrition (less than half [48%] completed all three modules).

Other evidence from evaluations of online interventions developed specifically for Aboriginal people, such as the Stay Strong App for setting goals relating to social and emotional well-being [14,15] and the iBobbly app for suicide prevention [16], indicates that electronic and Web-based therapeutic approaches may be feasible and acceptable to Aboriginal people. These treatment options may also have the potential to increase access to evidence-based therapies by overcoming barriers to health service access stemming from physical distance, cultural, or other barriers.

The We Can Do This online intervention is being developed as part of a larger project seeking to better understand and address deep concerns in Aboriginal communities about methamphetamine use through the development and trial of prevention and treatment strategies. Early quantitative and qualitative findings from the larger project indicate that access to health services does not currently match self-reported need and that barriers to health services exist, including stigma, shame, and a lack of culturally appropriate services. The online intervention may provide a pathway for people using methamphetamine to overcome such barriers to health service access or an alternative support option for people in the community.

### Objective

Our primary hypothesis is that participants who complete the We Can Do This online intervention will have significantly reduced days of methamphetamine use in the last 4 weeks at 1, 2, and 3 months from baseline compared with wait-listed participants with access to treatment as usual. Our secondary hypothesis is that the program will increase intended and actual help-seeking, including attendance at participating health services.

## Methods

### Trial Design

**Figure 1:**
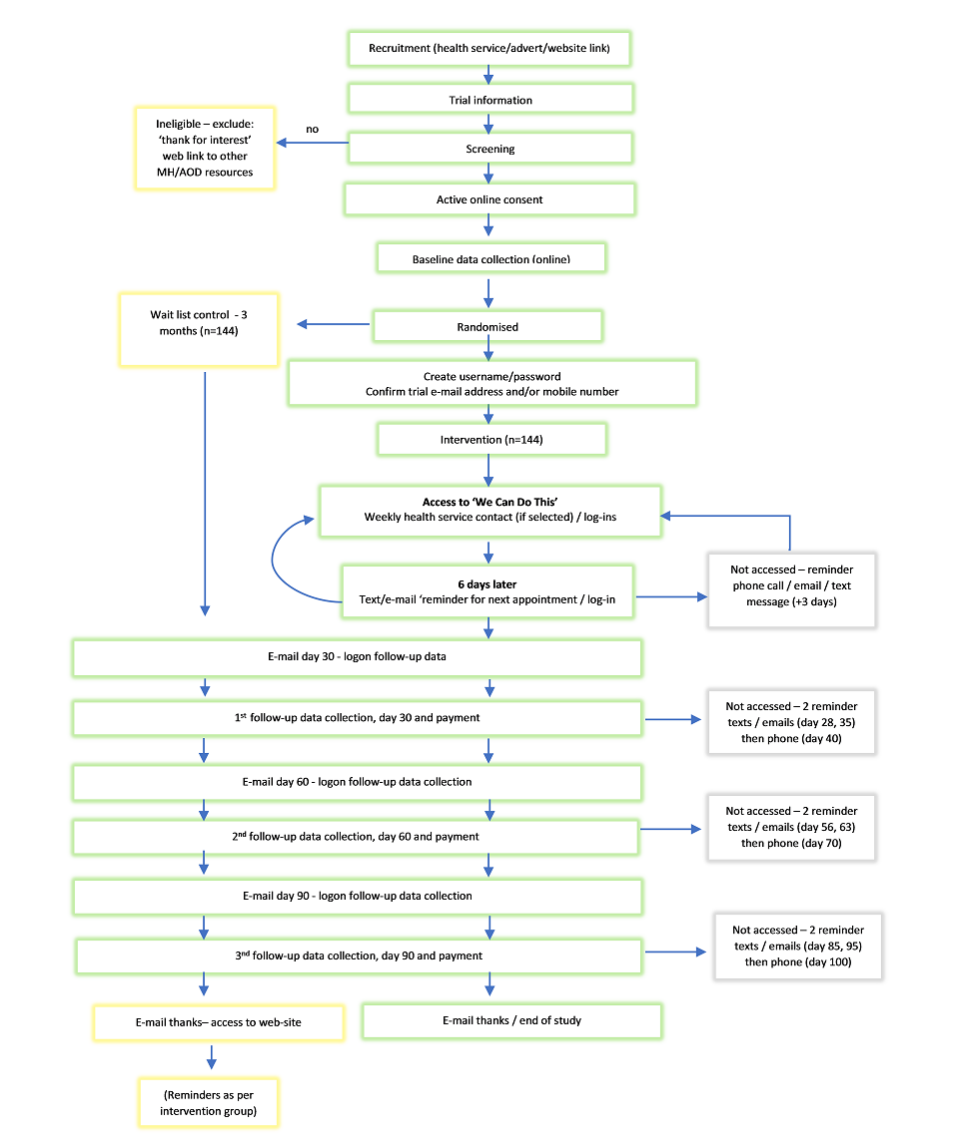
Flow of participants through the We Can Do This trial. AOD: alcohol and other drugs; MH: mental health.

A 2-group randomized controlled trial will be used. The intervention group will receive the We Can Do This online intervention with the option of telephone or face-to-face support provided by their local ACCHS staff. The wait-list control group will undertake the same assessments as the intervention group and have access to treatment as usual at the participating health services until the 3-month control period is complete, at which point they will receive access to the online intervention. They will receive reminders to log in but no further research assessments. Nonidentifiable use data will continue to be monitored. Treatment as usual will involve referral to online harm minimization information and access to alcohol and other drug counseling at the participating health service. The flow of participants through the trial is shown in Figure 1.

### Study Setting

The trial will take place in trial sites in geographically remote, regional, and urban locations in Australia. ACCHSs will provide telephone or face-to-face support to participants as required according to the research protocol.

### Participant Eligibility, Recruitment, and Consent

Participants will be Aboriginal and Torres Strait Islander people aged 16 years and over who have used methamphetamine at least weekly in the past 3 months. They will be recruited online through advertising and established social media channels in each of the trial sites. Recruitment will be restricted to those areas covered by ethical approval. Advertising material (posters, postcards) directing people to the project website will be available at participating sites and relevant alcohol and other drug services including needle exchange programs. Online screening questions will identify participants who meet the eligibility criteria and invite them to proceed to the online consent process. All participants will be presented with written plain-language information about the trial. The same information will be available online in audio-visual format, and the contact details of researchers and participating health service staff will be provided should participants wish to request further information or require assistance in any way. Participants will provide consent online. All participants will then complete the baseline assessment before being randomized to support via the online intervention or wait-list control (Figure 1).

### Randomization

The randomization scheme will be developed by a statistician who is not involved with study participants. Subjects will be randomly allocated to the online intervention or the wait list with 1:1 ratio. Random numbers will be generated using the statistical software Stata 14.0 or higher version (StataCorp LLC).

### Implementation

The online intervention may be accessed on any digital device (mobile phone, electronic tablet, or desktop computer) and requires continuous internet access to function during the trial. Video quality will adjust automatically according to available internet speed, and when the internet speed is too slow, a still image will appear and the participant will hear the audio track. Instructions will recommend that participants access the online intervention once a week for 6 weeks; however, they are free to access it as frequently as they wish during this time. Based on feedback from advisory groups, participants will not be required to complete modules in a set order but will be able to select modules based on preference. Participants may withdraw from the trial at any time. Data collected until that point will be included unless specifically requested otherwise. All participants will be paid Aus $20, $30, and $40 (US $14, $21, and $28) as reimbursement for their time completing the first, second, and third follow-up assessments, respectively. This payment will not be affected by participant level of engagement with the intervention.

Participants will have the option of receiving health practitioner support via weekly phone calls from trained staff at the participating health service in their geographical area. If this option is selected, participant contact details will be forwarded to the relevant health service, and they will receive an introductory phone call during which the health practitioner will explain their support role and schedule the weekly contact. Alternatively, participants will have the option to proceed without practitioner contact. In either case, participants will be prompted to consider the option of talking to someone at several points within the online intervention.

At the end of each module, printable summary pages of their inputs may be emailed to participants, their support people, and members of their Healing Circle as attachments according to participant preferences. With the participant’s permission, this summary may also be emailed to their allocated practitioner, if they have one. Participants will be sent an email or text to remind them to log in each week regardless of which modules have been completed. If all modules are completed early, participants may repeat modules. All online interactions will be monitored and inputs recorded but de-identified prior to analysis.

The online intervention has been developed so that a range of health practitioners may provide optional clinical support as an adjunct to the program. Health practitioners providing support will have received face-to-face training from the research team and a training manual with detailed instructions in the use of the online intervention and how it may be used as the basis for supportive conversations that consolidate the online intervention content. The manual contains examples of scripts to guide conversations. If issues arise in these conversations that fall outside the practitioners’ professional boundaries, they will have clear referral pathways available to them. Ongoing support for health practitioners will be provided throughout the trial by the research team and senior clinicians in each organization.

### Intervention

#### Web-Based Therapeutic Program

**Figure 2:**
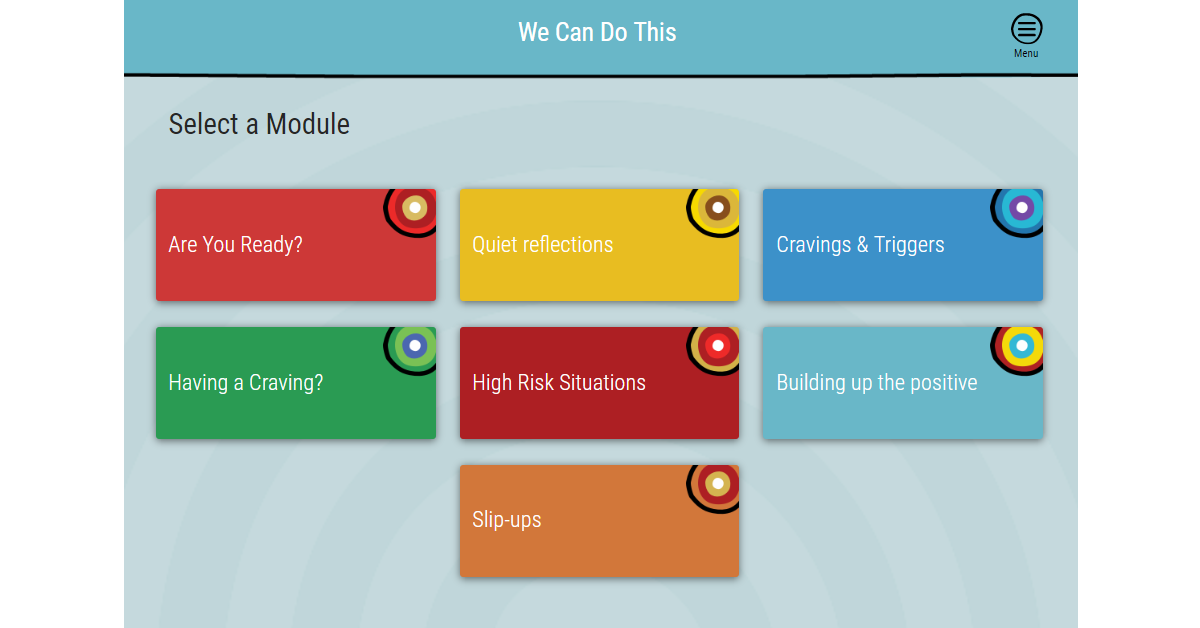
We Can Do This launch page.

The We Can Do This online intervention comprises 7 modules (Figure 2) that attempt to balance adherence to the strongest evidence-base, which points most clearly toward CBT and motivational interviewing treatment approaches, and advice from clinical and cultural advisors who steered the development toward ACT and narrative approaches. Achieving this balance required input from a range of experts. Three advisory groups were established: (1) a clinical advisory group comprising the research team and Aboriginal and non-Aboriginal clinicians working in drug- and alcohol-related fields, (2) a cultural advisory group comprising a senior representative from the diverse Aboriginal regions represented in the We Can Do This online intervention trial, and (3) a lived experience group comprising Aboriginal people with experience of methamphetamine use, either currently or in the past, who provided feedback on the online intervention acceptability and usability.

With a view to making the online intervention less text-heavy, films narrated and acted by Aboriginal actors that depict the lived experience of Aboriginal people are embedded within the online intervention to engage the participant and provide illustrations of the various issues covered. The films provide narratives that link modules together and an inviting way to engage with the content. The fictional characters depict different experiences with methamphetamine, as described in Textbox 1.

**Descriptions of fictional characters embedded within the We Can Do This online intervention.**

- Clinton used ice for 10 years but has not used for over 12 months. He has a supportive family, which is now his main focus, but he lost his relationship with his partner as a result of his use. He now has regular access to his young daughter and is motivated to keep healthy for her sake.
- Tanisha is a young woman whose use has been mainly recreational, with her boyfriend. She’s started noticing that she’s using more but it doesn’t feel as good anymore. In fact, she’s starting to wonder if it’s affecting her thinking. She’s also noticing that the drive to score ice has led her to do some things she regrets, including forgetting to pick up her little brother whom she takes care of.
- Joshua works hard and parties hard. He doesn’t really see his use as a problem, although his friends and family might beg to differ. Joshua has a full-time job in an office and has received a couple of warnings recently for turning up late. He’s also showing some physical effects from a lack of sleep, eating poorly and sometimes picking his skin.
- Aunty Rosie has a couple of nephews who are into ice; Rosie pulls no punches in letting them know what she thinks about it. She sees the ripple effect that using ice can have—not just on the person who uses but on the family, their friends, and the whole community.

Scripts for these films were developed from interviews undertaken for other parts of the larger Novel Interventions to Address Methamphetamine Use in Aboriginal and Torres Strait Islander Communities (NIMAC) project and informed by the findings of focus groups conducted in each of the 10 partner sites. Clinical and cultural advisors emphasized the importance of the visual aesthetic of the online intervention being both culturally appropriate and inviting, so artwork by an Aboriginal artist provides the backdrop for all modules. The modules are designed as stand-alone activities that do not have to be completed in a set order (Textbox 2). To increase accessibility for those with limited English literacy, set text within the modules is audio-recorded so that participants can choose to listen to rather than read the text. Some literacy will nonetheless be required to complete the program as at this stage participants’ own inputs are necessarily in written text.

**Figure 3:**
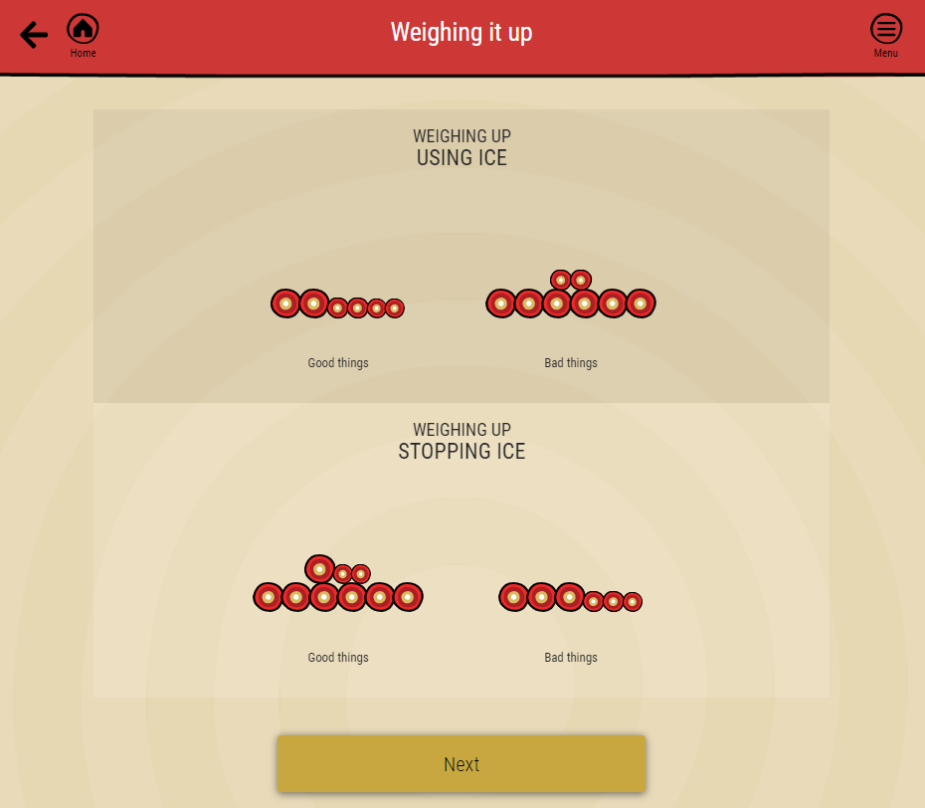
Weighing It Up visual summary.

At first log-in, participants will enter names of relevant support people into their Healing Circle. The participant may enter personal contacts or their own professional contacts with whom they would like to share their healing journey or whom they might contact for support. Helpline numbers and other relevant services will be automatically entered into this section of the online intervention and may be accessed at any time during use of the program from the menu icon in the top right-hand corner of each page, as shown in Figures 2 and 3. Some general instructions on the use of the online program are provided before the participant reaches the launch page, where they can access the 7 modules­­ (Figure 2).

#### Modules

##### Are You Ready?

This module is an online adaptation of the decisional balance exercise frequently used with motivational interviewing, although the exercise is commonly included in treatment manuals for substance use from different therapeutic modalities [17-19]. The exercise proceeds in 4 sections corresponding to good things about using ice, bad things about using ice, bad things about stopping ice, and good things about stopping ice. The choice of language—good versus bad things rather than the traditional good versus not so good—was made based on user feedback. A brief video illustrating relevant issues for one of the characters introduces each section. Within each section, participant is presented with up to 10 items (one per page) that they rate on a slide bar as not true for me, a little bit true for me, or true for me.

After rating the final item in each section, all items are presented in a list in order of relative weighting, and the participant can add further items to the list via a textbox. The weighting for each group of items is represented visually by a pile of stones, and the relative weighting given to each item is represented by the size of the stone (larger stones for those items rated as true; smaller stones for those rated as a little bit true). By the end of all 4 rating exercises, the good and bad things about using ice and stopping ice are represented visually by 4 piles of stones (Figure 3). Participants are then asked to identify the primary good thing about stopping ice that is most important to them and to pause to visualize the difference that stopping ice would make to this important area of their lives. Using mental imagery is known to promote goal-directed behaviors in a range of health-related domains [20,21] and has a long history as an important component of CBT [22]. Participants then have the option of scheduling a text message to themselves to arrive later in the week reminding them of this important motivator. The complete set of items selected, with relative weightings and all other inputs, is summarized in a PDF document that may be emailed or printed.

**We Can Do This modules and content.**

- Are You Ready? (10-20 minutes): aims to help participant resolve ambivalence and make a decision about what change (if any) they wish to make to their use.
- Cravings and Triggers (10-15 minutes): provides psychoeducation about the psychological and physiological processes involved in addiction. Exercises assist participant in making a behavioral change plan.
- Having a Craving? (5-20 minutes): behavioral experiment where participant rates the intensity of the craving; sets a timer for 10, 20, or 30 minutes; and is prompted to do other things and then sent a text message with a link back to the program after the specified time to rerate the craving.
- High-Risk Situations (10 minutes): participant identifies high-risk situations and makes a plan for coping with them with the option of receiving a supportive text message at their nominated high-risk time.
- Slip-Ups (10 minutes): reframes slip-ups as opportunities to learn. Participants are prompted to reflect on their experience and plan for next time.
- Quiet Reflections (5-15 minutes): participant may choose to engage with Rosemary Wanganeen’s approach to grief and loss, Joe Williams’ healing journey, or a simple mindful breathing exercise.
- Building Up the Positive (10 minutes): participant identifies the values that are most important to them and sets goals that will help them move closer to living a life that is aligned with their most important values.

##### Cravings and Triggers

The Cravings and Triggers module contains cognitive-behavioral exercises designed to assist the participant to identify their own triggers (situations, thoughts, and feelings that can lead to urges to use) and cravings (actual urges to use) with a view to making a plan for how to deal with them. The section begins with an informational video providing psychoeducation on the nature of addiction and withdrawal. This psychoeducation provides the rationale for subsequent exercises, which prompt the participant to reflect what cravings feel like for them and identify the internal (thoughts/feelings) and external (situations) triggers that lead them to use. The first exercise presents the participant with an image of a person, and they are invited to tap on various body parts to read about common symptoms of cravings, alongside strategies that can help to cope with that symptom. For example, tapping on the nose will bring up the message: “Smell can bring on strong cravings. You can interfere with them by smelling something else, lemon, coffee, soaps—anything that you find calming and not related to using ice.”

The next exercise is introduced with written information defining external triggers, followed by a video of Clinton describing triggers for him (eg, driving past a house where I used to party). The participant is then presented with a list of common external triggers that they can select from a list, with free text boxes to add detail. Aunty Rosie then suggests in a video that stopping ice use will require avoiding certain people and places, and the participant is asked to identify those triggers that may be simply avoided, including getting rid of any ice and equipment they may have left. Tanisha then introduces the idea of internal triggers, and the participant is again asked to select those that apply to them. Participant is then prompted to make a plan to reduce stress and practice identifying and responding constructively and compassionately to unhelpful thoughts using structured questioning. Again, all entries are summarized in a PDF to be printed or emailed.

##### Having a Craving?

This module is a brief behavioral experiment in which the participant is prompted to rate the intensity of their craving on a scale from 0 to 10 and set a timer for 5, 10, or 20 minutes, during which time they are instructed to do something else. Participant has the option of playing Tetris on their device, as there is some evidence that playing Tetris acts as visual cognitive interference and reduces the incidence and intensity of cravings in real-world settings [23]. When the timer finishes, the participant receives a text message prompting them to return to the exercise and rerate their craving, after which they can repeat the exercise or move on to another module.

##### High-Risk Situations

This module includes a series of questions designed to assist the participant to identify potential high-risk situations and make a plan to cope with them without using methamphetamine. Possible strategies include avoiding the situation altogether when possible, making a plan to leave if things get hard, rehearsing how to say no to people when drugs are offered, preparing by letting people know that they have decided not to use, having support people nearby/contactable, and reminding themselves of their primary motivation for reducing or stopping use.

##### Slip-Ups

In this module, slip-ups are differentiated from relapse and reframed as an opportunity for learning and getting stronger for next time. Unhelpful and helpful thoughts are articulated, and then a series of questions prompts the participant to reflect on how they were feeling and what they were doing and thinking to enable them to respond differently next time.

##### Quiet Reflections

This module contains a series of reflections and mindfulness exercises that may be selected from a launch page within the module. These reflections emphasize connection to culture, community, and identity and provide pathways for addressing culturally situated grief and loss and connecting with cultural programs. The module also contains a basic introduction to mindfulness via psychoeducation and a brief breathing exercise taken from Smout and Lazicki [19].

##### Building Up the Positive

Building up the Positive is an adapted version of the Bullseye Values Survey developed by Lundgren et al [24] and further popularized by Harris [25]. This exercise does not focus on drug use specifically and is intended to help the participant build a valued life outside of their use. In our adapted version, the participant is presented with three baskets labeled less important, important, and very important. Values appear on the screen and the participant sorts them into the appropriate basket. In the next stage of the exercise, those values rated as very important appear one by one and can be placed on a stylized image of a dartboard—close to the center if the participant feels they are living well in accordance with that value and further away from the center if they are not living in line with that value. Once all the values have been placed on the board, participant is encouraged to select a value placed further from the center to work on in the next section. Participant is then asked to write a specific, achievable action that would help them move closer to living out that value. This is followed by three questions: What would be the first step toward this goal? Who could help you do this? When will you do this? After nominating a day and time, participant has the option to send a text message to themselves reminding them to take the first step. Participant can then choose to work on another value, print their plan, or select another module.

#### Incorporation of Feedback From Focus Testing

The modules were focus tested in two phases. The first module developed (Are You Ready?) was focus tested with Aboriginal people with past or current experience of methamphetamine use recruited via organizational networks in urban and regional locations in South Australia and Victoria. Feedback from these groups was incorporated into the WBTP and informed the further development of subsequent modules. Later modules were focus tested with participants recruited via the same networks. Feedback was generally positive and supported the use of films to convey key messages. Participants found the content credible and useful and provided advice to improve the clarity of terminology, imagery, and usability. They also noted that while films are a powerful medium for conveying positive messages, the emotive nature of some stories could be triggering for some people, which prompted a greater focus on building safety measures into the online intervention—for example, ensuring timely access to face-to-face support when needed.

### Outcome Measures

#### Primary Outcome

The primary outcome measured will be the number of days the participant used methamphetamine during the treatment phase, assessed using questions from the Australian Treatment Outcome Profile (ATOP) [26]. The ATOP has been validated in treatment settings across Australia.

#### Secondary Outcomes

Differences in help-seeking will be assessed with the General Help-Seeking Questionnaire [27] and by the rate of referral and frequency, type, and duration of health service contact resulting from use of the online intervention. Other outcomes will include readiness to change (Readiness to Change Questionnaire) [28]; psychological distress (Kessler 10) [29]; poly-drug use during the past month (ATOP) [26]; severity of dependence (Severity of Dependence Scale) [30]; days out of role (referencing methamphetamine use rather than depression) [31]; and usability and acceptability (Internet Intervention Adherence Questionnaire [32], which includes questions about barriers and facilitators to using the program and optional free-text to report any criticisms or other feedback). Feasibility of the online intervention will be assessed overall by demographics of participants, general uptake and use of the online intervention (modules completed, time spent, etc), and number of participants who complete the program. Qualitative interviews with health service staff and at least two online intervention users will be conducted to explore positive and negative experiences.

### Sample Size and Power

Based on our previous research, we expect a baseline mean of 16 days methamphetamine use in the past 4 weeks [33]. We aim to detect a reduction to a mean of 8 use days in the past 4 weeks, as this will have a significant clinical impact in terms of reducing the risk of psychotic symptoms and violence (reduction from odds of 10-11 to 2-3) [33,34]. To detect this difference at each of the follow-up points with 90% power at a *P*<.01 level, we will require a sample of 100 participants per group. Based on our previous research, with assertive follow-up (ie, by phone as well as by email/text) we expect up to 30% attrition at follow-up, meaning that we will need to recruit 144 participants into each condition (intervention and treatment as usual) or a total of 288 participants. Treatment sites will be ACCHSs and other relevant services with Aboriginal clients.

### Data Collection and Management

Data will be collected online at baseline, and at 1, 2, and 3 months postbaseline. Participants will be invited to complete follow-up assessments online via a link sent by email and/or text message in the first instance and followed up by telephone thereafter. For analysis, participants will be assigned a unique patient identifier. De-identified data on rate of referral and frequency, type, and duration of health service contact resulting from use of the online intervention will be provided by the health service using the unique patient identifier allocated to each participant. Data collected online will be de-identified and uploaded automatically to a secure server managed by the primary sponsor (South Australian Health and Medical Research Institute) in accordance with strict research governance protocols [35]. Contact details are necessary for the successful operation of the online intervention but will not be stored with data collected for research purposes. Access to data will be limited to research staff named on the ethics protocol.

### Statistical Analyses and End Points

An intention-to-treat analysis will compare the primary and secondary outcomes for the intervention versus placebo conditions at 1, 2, and 3 months from baseline. Main effects will be evaluated using a mixed effect model that accounts for site-specific clustering effects.

### Safety and Security

The safety and security of participants is of paramount concern at all stages of the research process. To address issues of safety, the participant information sheet provided to participants will include emergency numbers of appropriate providers in the local area and the contact numbers of the relevant researchers. These will also be included online at the end of baseline and follow-up assessments. Adverse events or unintended effects of the trial reported by participants or clinicians involved in the trial will be assessed by the trial management committee and reported to the ethics committees and other parties as required.

### Ethical Approval

Approval from Aboriginal human research ethics committees at each of the participating sites and the Flinders University Human Research Ethics Committee has been obtained. Any modifications to the protocol will be approved by the trial management committee and communicated to all investigators, ethics committees, and the trial registry. The trial was registered at the Australian New Zealand Clinical Trials Registry [ACTRN 12619000134123p].

### Dissemination

Trial results will be reported to all participating sites and to the public via the Australian New Zealand Clinical Trials Registry and NIMAC websites. Topics suggested for presentation or publication in the peer-reviewed literature will be agreed upon by the chief investigators, and authorship will be based upon the four criteria outlined by the International Committee of Medical Journal Editors [36].

## Results

Funding for this trial was awarded from the National Health and Medical Research Council in 2016 (APP#1100696). Recruitment will commence in July 2019 and proceed for 12 months. Results are expected in early 2021.

## Discussion

Early identification of problematic methamphetamine use and access to timely and effective treatment options is critical to enhance opportunities for successful outcomes for people who use the drug; however, treating methamphetamine use and dependence is a health priority that has not been fully addressed. This will be the first trial of a culturally appropriate online therapeutic program for addressing methamphetamine use specifically among Aboriginal people. We believe that, if effective, the online intervention will provide benefits to Aboriginal people and health services in three main ways.

First, it will be a much-needed resource for Aboriginal people seeking to change their methamphetamine use. Importantly, it has the potential to reach those who face barriers to attending drug treatment and other health and social support services. The anonymous nature of an online intervention helps to overcome such barriers. Second, the online intervention incorporates basic referral mechanisms to professional support, such as the inclusion of links to and prompts to make contact with health services; therefore, it has the potential to link people using methamphetamine with appropriate face-to-face support. Third, the WBTP is a resource that may be used by counselors and other workers within ACCHSs and mainstream services who work with Aboriginal people. Access to alcohol and other drug counselors and registered psychologists is not always readily available; therefore, the WBTP will increase the range of clinicians who, with appropriate training, can deliver evidence-based and culturally appropriate therapeutic content to clients.

At present, barriers to accessing care for people using methamphetamine include physical distance from services, a lack of culturally appropriate health services, and the stigma associated with use. Online interventions have the potential to overcome these barriers and reduce methamphetamine-related harm for Aboriginal people, their families, and communities. Our trial will determine whether access to the internet is a barrier to use of such interventions. While the reliance on text has been reduced as far as possible, the study will also assess the extent to which this material is accessible to the intended user.

## Abbreviations

ACCHS: Aboriginal Community Controlled Health Service
ACT: acceptance and commitment therapy
ATOP: Australian Treatment Outcome Profile
CBT: cognitive-behavioral therapy
NIMAC: Novel Interventions to Address Methamphetamine Use in Aboriginal and Torres Strait Islander Communities
